# Partnered dance evokes greater intrinsic motivation than home exercise as therapeutic activity for chemotherapy-induced deficits: secondary results of a randomized, controlled clinical trial

**DOI:** 10.3389/fpsyg.2024.1383143

**Published:** 2024-06-19

**Authors:** Lise Worthen-Chaudhari, Patrick M. Schnell, Madeleine E. Hackney, Maryam B. Lustberg

**Affiliations:** ^1^NeuroArtsRx Laboratory, Department of Physical Medicine and Rehabilitation, College of Medicine, The Ohio State University, Columbus, OH, United States; ^2^Division of Biostatistics, College of Public Health, The Ohio State University, Columbus, OH, United States; ^3^Division of Geriatrics and Gerontology, Department of Medicine, Emory University, Atlanta, GA, United States; ^4^Center for Visual and Neurocognitive Rehabilitation, United States Department of Veterans Affairs, Atlanta, GA, United States; ^5^Center for Breast Cancer, Yale Cancer Center, Yale University, New Haven, CT, United States

**Keywords:** dance, neurorehabilitation, chemotherapy-induced neuropathy, neurologic dance training, self determination theory, intrinsic motivation

## Abstract

**Introduction:**

Dance has been proposed to support superior intrinsic motivation over non-dance forms of therapeutic physical activity. However, this hypothesis has yet to be evaluated empirically, particularly among populations living with neuropathology such as survivors of cancer with neurologic complications from chemotherapy treatment. Questions about motivation are relevant to clinical outcomes because motivation mediates neuroplasticity. We conducted this secondary analysis of a randomized-controlled study to begin to investigate the relationships between personal motivation and neurophysiologic effects of dance-based intervention for healthy aging among populations with neurologic complications of cancer.

**Methods:**

We measured motivation using the Intrinsic Motivation Inventory, a validated patient-reported outcome from the psychological approach of Self Determination Theory. We assessed intrinsic motivation, extrinsic motivation, and satisfaction with intervention within a randomized controlled trial of dance versus exercise designed to alleviate symptoms of chemotherapy-induced impairment. Fifty-two survivors of breast cancer with chemotherapy-induced neuropathy diagnosis and associated sensorimotor functional deficits were randomized (1:1) to 8 weeks of partnered dance or home exercise, performed biweekly (NCT05114005; R21-AG068831).

**Results:**

While satisfaction did not differ between interventions, intrinsic motivation was higher among participants randomized to dance than those randomized to exercise (*p* < 0.0001 at all timepoints: 2 weeks, 4 weeks, 6 weeks, and 8 weeks of intervention), as was extrinsic motivation at 2 weeks (*p* = 0.04) and 8 weeks (*p* = 0.01).

**Discussion:**

These data provide evidence that social dance is more motivating than the type of home exercise generally recommended as therapeutic physical activity. The results inform directions for future study of the effect of dance-based therapeutics on embodied agency, neuroplastic changes, and clinically-relevant neuropathic improvement.

## 1 Introduction

As early as 1976, clinician scholars proposed dance as capable of motivating individuals to engage in physical rehabilitation (Hecox et al., 1976). Implied in this proposal is the hypothesis that dance is more motivating than other forms of therapeutic physical activity (Dhami et al., 2015), yet an empirical test of this hypothesis remains to be conducted. To address the gap in knowledge, and as one starting point from which to explore the research topic of dance, embodied agency, neuroplasticity, and health-related outcomes, we directly tested the hypothesis that dance is more intrinsically motivating than other forms of therapeutic physical activity. This study was performed as a secondary analysis within a randomized, controlled clinical trial among breast cancer survivors with chemotherapy-induced neuropathy (CIN), a population in need of novel non-pharmacologic solutions to prevent and reverse neurological complications associated with cancer diagnosis and treatments.

The ability of a physical activity program to motivate participation among individuals with neuropathology is relevant to clinical outcomes because motivation mediates activity-based therapy participation and related neuroplasticity (Cramer et al., 2011). Furthermore, aspects of dance experiences related to embodied agency – such as autonomy, connectedness, and competence – mediate motivation (Ryan, 1982; Ryan and Deci, 2000). Therefore, we propose that motivation is relevant to the research topic because it represents a measurable, mechanistic link between embodied agency and neuroplasticity. In addition to testing the hypothesis that dance is more motivating than exercise as therapeutic physical activity, we review background information relevant to the relationships between embodied agency, motivation, and neuroplasticity within the context of dance-based treatment for neurological complications of cancer.

### 1.1 Neurological complications of cancer

Taxane-based chemotherapy is a lifesaving treatment for breast cancer, however, the effectiveness of these agents to control cancerous cell growth comes at a price. Taxane-based chemotherapy agents have been found to cause damage to healthy cells (e.g., nerve, brain, and cardiac) among up to 80% of survivors undergoing the treatment (Song et al., 2017; Lustberg et al., 2023). Thus, survivors treated with these lifesaving agents risk a Faustian bargain: the treatment to extend life causes debilitating neuropathology. Survivors seek non-pharmacologic interventions capable of reducing the impact of chemotherapy-induced conditions such as neuropathic pain (Swarm et al., 2013), cognitive impairment (“chemobrain” or “chemofog”) (Farahani et al., 2022), and premature brain aging (Koppelmans et al., 2012; Kesler et al., 2017; Du et al., 2022; de Ruiter et al., 2023).

Current standard-of-care (SOC) is to treat these symptoms with antidepressant medications, such as duloxetine, a pharmacological solution that masks, but does not rehabilitate, the sensorimotor deficits underlying symptoms (Worthen-Chaudhari et al., 2019; Mezzanotte et al., 2022). In the absence of adequate pharmacological solutions, physical activity has emerged as a leading non-pharmacological intervention capable of improving chemotherapy-induced impairments across functional domains of the human system (Campbell et al., 2019; Kleckner et al., 2021). Physical activity is defined by the American College of Sports Medicine (ACSM) as muscular work that increases caloric expenditure substantially over resting levels of energy expenditure (ACSM, 2021) and can take many forms.

### 1.2 Types of physical activity studied as physical medicine

Exercise and dance are two different forms of physical activity that have been studied as potential activity-based therapy. Exercise is sometimes misunderstood as synonymous with physical activity but is defined as a subset of physical activity that is planned, structured, repetitive, and performed for the purpose of improving physical fitness (ACSM, 2021). Currently, exercise is the most studied form of physical activity through which to treat the neurologic complications of cancer. Multi-modal exercise was shown to improve sensation and motor function (Streckmann et al., 2014; Zimmer et al., 2018; Kleckner et al., 2021) while partnered strength training exercise improved strength (Winters-Stone et al., 2016) among survivors. Despite this promising evidence, survivors report that conventional exercise can be challenging to motivate themselves to participate in (Frikkel et al., 2020; Kim et al., 2020). Therefore, motivation to participate might represent a critical weakness of exercise as a therapeutic physical activity option.

Dance is another form of physical activity that has been studied as physical medicine for neural deficits, also known as neurorehabilitation. Previously, we reported preliminary evidence that partnered Tango dance improved postural control among survivors with neuromotor deficits (Worthen-Chaudhari et al., 2019). Outside of study in cancer populations, dance activity was reported to increase Brain-Derived Neurotropic Factor (BDNF), important for learning and memory in the aging brain (Rehfeld et al., 2018), and decrease inflammatory biomarkers associated with Alzheimer’s Disease (Wharton et al., 2021). While dance can be characterized as exercise when practice is planned, structured, repetitive, and performed intentionally for fitness goals (e.g., aerobic dance performed for cardiovascular fitness), we and others have defined dance as a form of physical activity than is distinguishable from conventional exercise (Rehfeld et al., 2018; Worthen-Chaudhari et al., 2019; Blumen et al., 2023). Elements of the dance experience that are intentional versus incidental hold the key to differentiating between dance that does or does not meet the definition of exercise. For example, dance performed as a social, musical, and artistic endeavor – such as Argentine Tango, disco dancing, or Irish line dancing - does not fit neatly within the definition of exercise (Worthen-Chaudhari et al., 2019). While individuals performing these dances experience physical fitness improvements, fitness is incidental to the endeavor. Mechanistic aspects of social, musical, and artistic dance include neural synchrony (Basso et al., 2021), chronoception, auditory-motor entrainment, spatial cognition (Brown et al., 2005; Chauvigné et al., 2014), and interpersonal coordination characterized as activating regions of the brain involved in working memory, tactile motion processing, and social reward (Chauvigné et al., 2017, 2018). If prior assumptions are correct, then another key difference distinguishing dance from exercise is motivation (Hecox et al., 1976; Dhami et al., 2015).

### 1.3 Motivation mediates neuroplasticity

Neuroplasticity has been defined, broadly, as the ability of living nervous systems to remodel in terms of structure, connectivity, and function in response to stimuli that might be external and/or internal (Cramer et al., 2011). This concept overlaps with the mathematical dynamical systems approach which predicts that living systems self-organize across domains of function in response to experiential stimuli (Fogel and Thelen, 1987; Thelen et al., 2001). A 2009 NIH working group summarized the following themes for clinically-relevant neuroplasticity: (a) experience dependence, (b) time sensitivity, (c) context dependence and (d) the importance of motivation and attention as mediators (Cramer et al., 2011). We previously reviewed ways that dance-based activity has the potential to stimulate neuroplasticity through these common themes (Worthen-Chaudhari, 2011). The current report focuses on the last common theme proposed by the working group: the importance of motivation as a mediator of neuroplastic outcomes. Given the importance of motivation for outcomes, more study is needed to characterize motivational aspects of candidate physical activity-based therapies.

### 1.4 Motivation is measurable

Definitions from the psychological approach of Self Determination Theory can help us to characterize the motivation invoked by different forms of physical activity. This framing differentiates between motivation oriented in intrinsic versus extrinsic factors. Intrinsic motivation refers to engaging in an endeavor because it is inherently interesting or enjoyable. In contrast, extrinsic motivation refers to engaging in an activity because the endeavor leads to a separate desired outcome (e.g., symptom relief, future health benefits, financial compensation). Importantly, Self Determination Theory hypothesizes that intrinsic motivation is superior to extrinsic motivation for the purpose of promoting healthy and sustained self-regulation of behavior (Deci et al., 1994; Ryan and Deci, 2000).

According to these definitions, exercise is fundamentally extrinsically motivated because exercise is defined as being performed for the future goal of improved physical fitness (ACSM, 2021). This is not to say that exercise cannot be interesting or enjoyable, because it can. This means that the intention of exercise is to attain a fitness goal and any interest or enjoyment in the activity is incidental. Studies have used the Self Determination Theory assessment tools to characterize exercise as extrinsically motivated among neurologically-intact populations (McAuley et al., 1989, 1991; McAuley and Tammen, 1989; Tsigilis and Theodosiou, 2003; Cocca et al., 2022) but study is lacking among individuals with neuropathology. Moreover, no study to date has compared intrinsic versus extrinsic motivational characteristics of exercise versus other forms of physical activity for the purpose of neurorehabilitation design. Given the relevance of motivation to neuroplasticity (Cramer et al., 2011), such an evaluation is needed.

### 1.5 Motivation is mediated by aspects of embodied agency

We speculate that motivation is mediated by select aspects of embodied agency in ways that move us out of extant definitions and require futurizing. Specifically, we posit that existing psychological definitions of autonomy, competence, and relatedness (APA Dictionary of Psychology, 2024) represent components of embodied agency. Embodied agency can be conceptualized as one’s sense of agency, or the feeling of being in charge of our actions (Moore, 2016), and specifically to the corporeal origins of that sense or feeling (EMBODIED Definition and Meaning, 2024). Autonomy describes the capacity of the embodied agent to make their own decisions and actions, without being told what to do. Competence refers to the ability of the embodied agent to cope with specific problems effectively. And relatedness describes the connection of the embodied agent to the environment as well as other agents in their environment. Self Determination Theory posits that personal motivation is mediated by autonomy, competence, and relatedness (Ryan, 1982), and we draw the conclusion that the measurable construct of motivation is mediated by embodied agency. More research of embodied agency is needed to understand all the ramifications of the concept. Centering agency in the body is a complex and fascinating conceptualization that promises to advance understanding of action, cognition, and physical medicine-based intervention.

Therefore, we sought to establish fundamental knowledge about motivational characteristics of dance versus exercise as therapeutic activity. Survivors of breast cancer with chemotherapy-induced neuropathy (CIN) were randomized to one of two interventions: partnered dance (dance) versus an evidenced-based exercise program (exercise). We hypothesized that intrinsic motivation would be higher among survivors randomized to dance than to exercise. If the hypothesis is proved true, then this study will represent the first empirical data supporting the prior assumption that dance is more interesting/enjoyable than exercise as therapeutic physical activity (Hecox et al., 1976; Dhami et al., 2015). As an exploratory measure, we examined extrinsic motivation to provide insight into the relative value attributed by survivors to dance or exercise in terms of alleviating their neurologic symptoms. Finally, as an additional exploratory variable, we compared satisfaction with intervention between interventional groups. Satisfaction is commonly queried in survivorship support settings to judge success of activity programming. Therefore, existing therapeutic programs may have extant data on satisfaction that might be repurposed to evaluate motivation; we report satisfaction with intervention alongside intrinsic and extrinsic motivation to begin to compare between the three measures.

## 2 Methods

This study was approved by the Institutional Review Board of The Ohio State University and is described in full in the clinicaltrials.gov registry item NCT05114005 (registered 08/15/2021) as well as in the protocol report by Lantis et al. (2023).

### 2.1 Participants

Fifty-two survivors of breast cancer (BC) with chronic chemotherapy induced neuropathy (CIN) were randomized 1:1 into dance (*n* = 26) or exercise (*n* = 26) intervention arms [51F/1M; age mean (SD) = 61 (9.7) years old; years since last taxane-based agent exposure = 2.9 (2.1)]. Participant demographics per group are summarized in Table 1.

**Table 1:** Participant demographics.

**Demographic variable**	Descriptor	**Tango**	**Home exercise**	**Overall**
Age (years)	MeanMedian	63.6 (8.37)63.8 [41.5, 78.7]	58.9 (10.4)60.6 [40.8,80.1]	61.2 (9.65)63.2 [40.8, 80.1]
Sex	Female/Male	25/1 (96%/4%)	26/0 (100%/0%)	51/1 (98%/2%)
BMI	MeanMedian	31.7 (7.46)30.4 [20.2, 53.5]	33.1 (8.97)33.2 [20.4, 52.0]	32.4 (8.20)31.2 [20.2, 53.5]
Race	AsianBlackWhite	1 (3.8%)2 (7.7%)23 (88.5%)	0 (0%)1 (3.8%)25 (96.2%)	1 (1.9%)3 (5/8%)48 (92.3%)
Ethnicity	NOT Hispanic or Latino	26 (100%)	26 (100%)	52 (100%)
Years since last taxol	MeanMedian	2.74 (1.78)2.57 [0.416, 5.98]	3.15 (2.45)2.92 [0.153, 7.95]	2.94 (2.13)2.67 [0.153, 7.95]

### 2.2 Interventions

Both interventions were designed to be completed within 1.25 h of participant time, at a frequency of twice per week over an 8-week period. Both interventions are detailed in Lantis et al. (2023) and summarized in Table 2.

**Table 2:** Summary of physical activity design elements per intervention.

**Intervention design component**	**Tango**	**Home exercise**
Recommended standard-of-care?	No	Yes
Was the movement practice musically entrained?	Yes	No
Was the movement practice socially engaged?	Yes	No
Was the intervention delivered in a group or individually?	Group	Individual
Was shared decision making practiced between participant and staff?	Yes	Yes
Total participant time commitment per intervention session	≤1.25 h	≤1.25 h
Target physical activity dose/class or session	20 min	45 min
Physical activity focus	Ease and technique of movement (balance skill, partnering, musicality)	Conditioning (balance skill, aerobic endurance, strength building)

The partnered dance (dance) intervention consisted of partnered, Adapted, Argentine Tango dance (AdapTango) performed to traditional Argentine Tango music (Hackney and McKee, 2014; Worthen-Chaudhari et al., 2019). This dance technique has been reported to improve balance, executive function, and biomarkers of inflammation for individuals with neurodegeneration including Parkinson Disease [citation] and prodromal AD (Wharton et al., 2021). AdapTango was delivered via in-person instruction by a former professional dancer and certified medical exercise specialist (Worthen-Chaudhari) who was certified to teach AdapTango technique by its developer (Hackney). Dance aesthetic pedagogical points (Fontanesi and DeSouza, 2021) included: musicality, ease of partnering, graviception (Fitze et al., 2024), clean execution of technique (e.g., weight shifts in time with the music), and coordination of breathing with voluntary motion. Patient-reported outcome data were recorded using the REDCap platform. We monitored participants’ self-reported rate of exertion using the 20 point Borg Rating of Perceived Exertion scale, aiming for ratings that achieved no higher than “somewhat hard” (i.e., ≤13 out of 20 on the Borg scale) in either the physical or mental domain (Lantis et al., 2023). We partnered 1–6 survivors per session with an equal number of volunteers without neuropathy who were trained previously in fall prevention and basic AdapTango partnering by Hackney and Worthen-Chaudhari.

The home exercise (exercise) intervention consisted of conditioning-focused physical activity [e.g., balance skill, aerobic conditioning, strengthening, flexibility (i.e., nerve glides)] previously found to improve CIN symptoms and function among cancer survivors (Kleckner et al., 2018; Zimmer et al., 2018). This intervention was assigned as a home exercise program: qualified staff taught the exercise sequences in person with each participant, individually, allowing time for clarifications as needed, before participants were expected to reproduce exercises safely and independently at home. We addressed safety features (e.g., use of a wall, chair, or surface for safety) and provided instructional aids (e.g., pictorial aids and YouTube channel videos to demonstrate safe performance technique) (Lantis et al., 2023). Patient-reported outcome data were recorded using the MyCap application installed on participants’ phones. Social and programmatic support was provided weekly by research staff, via participant’s preferred form of contact (i.e., phone call, email, text message, MyCap message), to encourage, troubleshoot, and increase challenge point of the program through a shared decision-making process (Playford, 2014) between qualified staff and participants.

### 2.3 Outcomes

#### 2.3.1 Intrinsic and extrinsic motivation

We administered subscales of the Intrinsic Motivation Inventory (IMI) to test for both intrinsic (primary outcome) and extrinsic motivation (exploratory outcome). The subscales of the IMI were developed and validated by several groups of scientists starting in the late 1980s (McAuley et al., 1989, 1991; Ryan et al., 1991; Deci et al., 1994; Ryan and Deci, 2000; Tsigilis and Theodosiou, 2003; Intrinsic Motivation Inventory, 2024).

#### 2.3.2 Intrinsic motivation (primary outcome)

The 6 item Interest/Enjoyment subscale of the IMI represents intrinsic motivation in an activity just performed as a possible score of 6 to 36 with 6 = most; 18 = neutral; 36 = least interesting/enjoyable. Specific items queried include whether the activity just performed was: enjoyable, enjoyed, boring (reverse order), interesting, fun, and able to hold the participant’s attention.

#### 2.3.3 Extrinsic motivation (exploratory measure)

The 7-item Value/Usefulness subscale of the IMI represents extrinsic motivation in an activity just performed as a score of 7 to 49 wherein 7 = most; 24 = neutral; 49 = least valuable/useful. Specific items queried include whether the activity just performed was: one the participant would be willing to do again, helpful for neuropathy symptoms, beneficial, important, helped the participant to cope with neuropathy symptoms, and valuable as therapeutic for neuropathy symptoms.

#### 2.3.4 Satisfaction (exploratory measure)

We captured satisfaction with intervention after each class using a 7 pt. Likert scale and prompt for feedback about what did/did not work per class. Satisfaction feedback was used to improve future sessions for both interventions. We administered the satisfaction with intervention measure (1 item) immediately before intrinsic then extrinsic motivation measures. The order of instrument administration did not change between timepoints.

### 2.4 Procedures

Participants were recruited through a process in which we mined electronic medical records for eligible individuals and worked with treating oncologists to contact those eligible, as well as through direct referral from clinicians, and flyers posted in the community.

Timepoints of instrument assessment occurred bi-monthly at the end of physical activity session in intervention week 4, 8, 12, and 16. During in-person research sessions, we used the Research Electronic Database Capture (REDCap) application, installed on laboratory laptops, to document satisfaction and motivation outcomes. For remote data capture, we used the MyCap application, a REDCap platform installed on participant’s smartphones, to document adherence, IMI and satisfaction ratings, and adverse events.

### 2.5 Statistical analysis

A linear mixed model was applied to log-transformed scores. Data were assessed regarding normality and appropriate descriptive statistics were calculated. Results are reported on original scales. Cronbach’s alpha was computed for motivation measures in combined study arms, separately for each timepoint.

## 3 Results

### 3.1 Primary outcome

**Figure 1:**
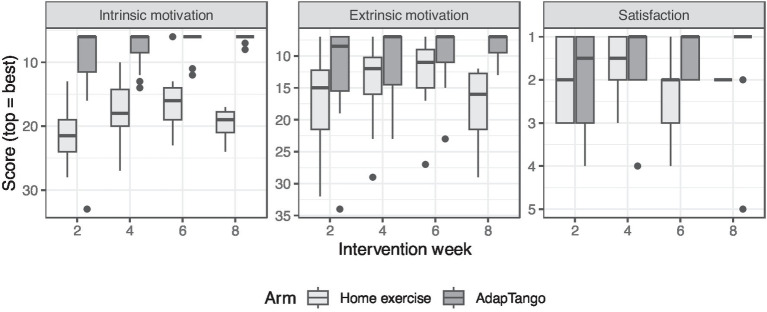
Box plots of motivation and satisfaction by week. Middle lines represent median values while tops and bottoms of boxes represent 1st and 3rd quartiles. Ends of whiskers represent the most outlying points within 1.5 inter-quartile range (IQR) of the 1st or 3rd quartiles. Dots represent outliers beyond 1.5 IQR. Intrinsic motivation possible score range is 6–36 (6 = best). Extrinsic motivation possible score range is 7–49 (7 = best). Satisfaction possible score range is 1–7 (1 = best).

Intrinsic motivation was higher among those randomized to the dance intervention for all timepoints tested (*p* < 0.0001 all comparisons, Figure 1 and Table 3).

**Table 3:** Intrinsic motivation values (median [IQR]) per intervention and timepoint (*p* < 0.0001 all) where possible scores range from 6 to 36 (6 = most; 18 = neutral; 36 = least interesting/enjoyable).

Intrinsic motivation	Wk4 median [IQR]	Wk8	Wk12	Wk16
Dance	6 [6–11.5]	6 [6–8.5]	6 [6–6]	6 [6–6]
Exercise	21.5 [19–24]	18 [14.2–20]	16 [14–19]	19 [17.8–21]
Between group comparison *p*-value	p < 0.0001	p < 0.0001	p < 0.0001	p < 0.0001

### 3.2 Exploratory outcomes

Extrinsic motivation was high in both arms with a statistically significant difference at weeks 2 (*p* = 0.04) and 8 (*p* = 0.01). Grouped across timepoints, dance medians were 7–8.5, first quartile 7–7, third quartiles 11–15.5 while exercise medians were 11–16, first quartiles 9–12.8, third quartiles 15–21.5. Cronbach’s alpha is reported in Table 4.

**Table 4:** Cronbach’s alpha values for intrinsic and extrinsic motivation subscales.

Measure	Wk4 alpha (95% CI)	Wk8	Wk12	Wk16
Intrinsic Motivation subscale	0.939 (0.892–0.964)	0.846 (0.650–0.947)	0.859 (0.685–0.943)	0.940 (0.882–0.984)
Extrinsic motivation subscale	0.906 (0.766–0.959)	0.894 (0.809–0.949)	0.877 (0.615–0.953)	0.936 (0.723–0.988)
Sample per timepoint	28	29	22	15

Similarly, satisfaction with intervention was high in both arms with no statistically significant differences at any timepoint (*p* = 0.07 to 0.71). Grouped across timepoints dance medians were 1–1.5, first quartiles 1–1, third quartiles 1–3 while exercise medians were 1.5–2.5, first quartiles 1–2, third quartiles 2–3.

## 4 Discussion

The proposed hypothesis was supported: partnered dance was more intrinsically motivating than home exercise as therapeutic activity among this cohort of survivors with BC and measurable CIN-related sensorimotor deficits. More research is needed to establish whether this effect is reproducible and generalizes to other populations living with neuropathologic impairments. However, the data provide a starting point from which to examine dance as Neurologic Dance Training practice with unique ability to motivate physical activity engagement that stimulates neuroplasticity relevant to rehabilitation and healthy aging. To describe these aspects of the dance intervention studied in a way that facilitated translation between the domains of dance and neurorehabilitation scholarship, we apply the Rehabilitation Treatment Specification System (RTSS) (Hart et al., 2019; Van Stan et al., 2019; Zanca et al., 2019).

**Figure 2:**
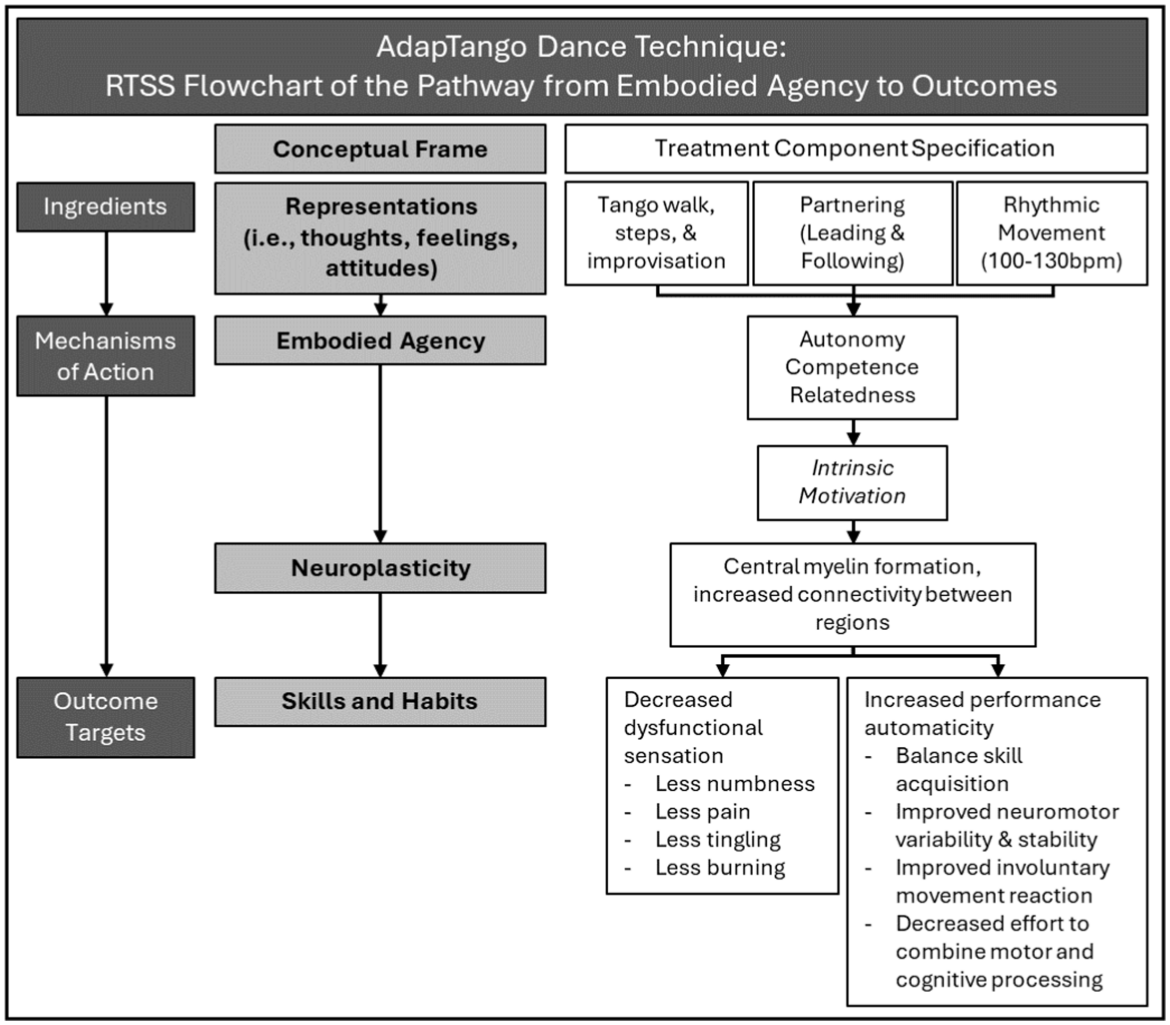
Rehabilitation Treatment Specification System (RTSS) schematic of the mechanistic pathway through which representations of embodied agency might lead to improved clinical outcomes. The variable measured for this report *– intrinsic motivation –* is shown in *italics.*

The RTSS provides a taxonomy through which to document evidence-based neurorehabilitation interventions in ways that are reproducible, aligned with treatment theory, and facilitate future study and implementation. Interventional components are articulated in terms of conceptual frame and pragmatic specification. Once specified, the intervention can be diagrammed as treatment component ingredients (e.g., Tango walk performed in a partner practice hold to tango music) that drive mechanistic actions (e.g., intrinsic motivation), toward a clinical outcome target (Figure 2). Articulating novel interventions in this way, and in such detail, increases the chances of success for health and research professionals who may seek to study and/or implement interventions in future. Tracing the causality between treatment components and outcomes in Figure 2 makes clear the potential to effect physical skills and habits through treatment ingredients focused on internal representations of affective and cognitive processes - such as thoughts, feelings, and attitudes during movement performance.

There are several ways in which intrinsic motivation in dance might drive measurable aspects of neuroplasticity such as central myelin formation (Lakhani et al., 2016; Faw et al., 2021) and increased structural connectivity between brain regions (Thumuluri et al., 2022). Firstly, intrinsic motivation might drive neuroplasticity via increased activity dose participation: participants could attend more sessions. More study is required to assess these results in the context of factors affecting attendance such as older age, which has been reported to increase adherence to supervised exercise (Courneya et al., 2012).

Secondly, intrinsic motivation might mediate the way that the human dynamical system attends to an endeavor. Engagement in dance-based endeavors might strengthen the coupling of goals to actions, which should improve performance automaticity as predicted by the OPTIMAL (Optimizing Performance through Intrinsic Motivation and Attention for Learning) theory of motor learning (Wulf and Lewthwaite, 2016). As but one example, artistic engagement might engage the motor and cognitive systems in unique combinatorial ways that drive motor-cognitive integration, with consequences for health outcomes. As another example, artistic engagement might serve to increase level of effort and associated clinical outcomes. Prior research characterized attention in inpatient movement therapies in terms of clinician observed “level of effort” and found that high level of effort improved outcomes at discharge (Seel et al., 2015).

A third avenue through which intrinsic motivation to participate in dance modulates outcomes has to do with relatedness: dance might support psychosocial needs through providing comfort during physical training. Qualitative research among survivors has identified the theme of comfort as a key psychosocial need and has identified social connection and personal expression as two specific forms of comfort known to support stress management associated with cancer diagnosis and treatment (Williams and Jeanetta, 2016; Kim et al., 2020; Durosini et al., 2021). Dance performed in a group or with a partner provides opportunity for exploring social connection with other survivors while dance forms that incorporate creative and/or artistic improvisational elements likely support personal expression (Hecox et al., 1976; Dhami et al., 2015). Accordingly, dance forms that provide opportunity for enhanced relatedness – that takes the form of social connection and/or personal expression – are most likely to provide comfort in ways that support psychosocial needs of survivors and facilitate activity adherence (Kim et al., 2020).

A fourth aspect of intrinsic motivation to consider involves the neural regulation and role of the neurotransmitter dopamine and the role of dopamine in play behavior. A combination of pre-clinical and human neuroimaging studies have found that dopamine is involved in motor control, motor learning, and synaptic plasticity (Cousins and Salamone, 1996; Molina-Luna et al., 2009; Hosp et al., 2011; Da Silva et al., 2018; Koshimori et al., 2019). Because circulating dopamine is upregulated by both social (Gunaydin and Deisseroth, 2014) and musical (Salimpoor et al., 2015) experiences, dance activities that include social and musical engagement are likely to increase available dopamine in the human system in ways that potentiate skill acquisition and associated neuroplasticity. One area of emerging research with potential to elucidate the role of dopamine in intrinsic motivation, specifically, is the area of play-based learning (Kellman and Radwan, 2022). Play has been defined as fundamentally intrinsically motivated in terms of being “done for its own sake” (Burghardt, 2005) and dopamine has been implicated as a key neurotransmitter in such behavior (Siviy and Panksepp, 2011). Future research should consider commonalities in neurotransmitter activity between feelings of embodied agency and play behavior. It is possible that dance provides an avenue for physical play behavior in adults, and that resulting dance-based play invokes embodied agency, intrinsic motivation, and clinically-relevant neuroplasticity.

Indeed, the variety of avenues through which the system might spontaneously, playfully, and/or intentionally engage within the AdapTango intervention may present opportunity for participants to choose and vary the nature of their personal engagement in the activity. While dancing, an individual can tune their attentional focus to different aspects of performance (e.g., social, musical, physical) in any combination that they choose, potentially even shifting between these foci in an improvisatory way. Affording choice within physical activity interventions provides autonomy support and has been shown to facilitate motor learning (Wulf et al., 2014; Lewthwaite et al., 2015; Wulf and Lewthwaite, 2016). Therefore, the variety of avenues through which AdapTango might motivate the human system to engage represent unforced opportunities for personal choice that could prove to be contextual factors influencing neuroplasticity. Lastly, these aspects of motivated performance may mediate neuroplasticity directly, as well as create a mechanistic loop that enhances sense of autonomy, competence, and/or relatedness during performance of the endeavor, which further enhances motivation and associated neuroplasticity, looping back to re-engage the performer’s sense of autonomy, competence, and relatedness. More study is needed to identify feedback loops that might be generated through arts-based therapeutic activity and the role of such reinforcing loops in clinically-relevant neuroplasticity.

More study is also needed of the interplay between health outcomes, intrinsic motivation, and the exploratory measures we report of extrinsic motivation and satisfaction. Dance was higher in extrinsic motivation than exercise at 2 and 8 weeks of intervention, in addition to being higher in intrinsic motivation at all timepoints tested. Despite this profile of higher motivation, we found no evidence for a difference in satisfaction between interventions. It is possible that the satisfaction ratings commonly gathered to evaluate therapeutic programming do not reflect the more mechanistic concept of motivation. More research is needed of these internal representations of motivation and satisfaction regarding their ability to predict neuroplasticity and associated neurorehabilitation outcomes.

Like any scientific study, this one is limited. For instance, we are limited in that we cannot differentiate between the various aspects of engagement leading to more intrinsic motivation in the dance versus the exercise intervention. Intrinsic motivation might be more attributable to social, musical, physical, cognitive, interoceptive, autonomic, artistic, or other aspect of engagement unique to the dance activity. Moreover, we are limited in characterizing engagement across the cohort because any pattern of engagement is likely to be patient-specific and to vary with time. Embodied agency represents one promising umbrella concept under which these varied avenues of dynamic system engagement might be represented and studied. Another limitation of this study is the poor diversity represented within the study cohort. Future study will address this issue and increase representation across survivors of different racial and ethnic backgrounds. Lastly, we did not study neuroplasticity directly so cannot confirm that the differences in intrinsic motivation led to corresponding differences in neuroplasticity. This is an area of future study, however, that the current research supports examining.

These data provide evidence that social dance is more intrinsically motivating than the type of home exercise generally recommended as therapeutic physical activity. The results inform directions for future study of the effect of dance-based therapeutics. More study is needed to elucidate relationships between dance-based intervention, embodied agency, motivation, neuroplastic changes, and clinically-relevant neuropathic improvement.

## Data availability statement

The raw data supporting the conclusions of this article will be made available by the authors, without undue reservation.

## Ethics statement

The studies involving humans were approved by The Ohio State University Institutional Review Board. The studies were conducted in accordance with the local legislation and institutional requirements. The participants provided their written informed consent to participate in this study.

## Author contributions

LW-C: Conceptualization, Data curation, Formal analysis, Funding acquisition, Investigation, Methodology, Project administration, Resources, Software, Supervision, Validation, Visualization, Writing – original draft, Writing – review & editing. PS: Conceptualization, Data curation, Formal analysis, Funding acquisition, Methodology, Visualization, Writing – original draft, Writing – review & editing. MH: Conceptualization, Formal analysis, Funding acquisition, Investigation, Methodology, Supervision, Writing – original draft, Writing – review & editing. ML: Conceptualization, Data curation, Formal analysis, Funding acquisition, Investigation, Methodology, Project administration, Resources, Supervision, Validation, Visualization, Writing – original draft, Writing – review & editing.
